# BrainK for Structural Image Processing: Creating Electrical Models of the Human Head

**DOI:** 10.1155/2016/1349851

**Published:** 2016-05-16

**Authors:** Kai Li, Xenophon Papademetris, Don M. Tucker

**Affiliations:** ^1^Electrical Geodesics, Inc., 500 E. 4th Avenue, Eugene, OR 97401, USA; ^2^Yale University, New Haven, CT 06520, USA

## Abstract

BrainK is a set of automated procedures for characterizing the tissues of the human head from MRI, CT, and photogrammetry images. The tissue segmentation and cortical surface extraction support the primary goal of modeling the propagation of electrical currents through head tissues with a finite difference model (FDM) or finite element model (FEM) created from the BrainK geometries. The electrical head model is necessary for accurate source localization of dense array electroencephalographic (dEEG) measures from head surface electrodes. It is also necessary for accurate targeting of cerebral structures with transcranial current injection from those surface electrodes. BrainK must achieve five major tasks: image segmentation, registration of the MRI, CT, and sensor photogrammetry images, cortical surface reconstruction, dipole tessellation of the cortical surface, and Talairach transformation. We describe the approach to each task, and we compare the accuracies for the key tasks of tissue segmentation and cortical surface extraction in relation to existing research tools (FreeSurfer, FSL, SPM, and BrainVisa). BrainK achieves good accuracy with minimal or no user intervention, it deals well with poor quality MR images and tissue abnormalities, and it provides improved computational efficiency over existing research packages.

## 1. Introduction

With the simple recording of the electroencephalogram (EEG), the brain's electrical activity can be measured with millisecond temporal resolution at the head surface. Dense array EEG (dEEG) systems now allow up to 256 channels to be applied quickly with full coverage of the head, assessing the fields from the basal as well as superior cortical surface [1, 2]. The cortex, with its laminar neural organization and with locally synchronous activity stemming from its columnar organization, is the primary generator of the far fields measured by head surface EEG [3]. Cortical sources can be modeled as point dipoles, and their contribution to surface activity can be reconstructed through electrical source analysis. The ambiguity of the inverse estimation in electrical source analysis can be minimized if the precise locations and orientations of the cortical sources are well specified. To a first approximation, the source dipoles can be assumed to be oriented perpendicular to the cortical surface, consistent with the orientation of the pyramidal neurons and cortical columns.

To model cortical sources with these properties, accurate cortical surface extraction is a key challenge. Furthermore, the volume conduction of the electrical potentials, from the cortex to the head surface, must be specified through characterizing the conductivity of each tissue compartment. The skull is the primary resistive medium in the head, and it must be modeled, preferably with bone density values from CT. Because the electrical boundary effects of the volume conduction are affected by discontinuities in current paths, for example, caused by holes in the skull (optical canals and foramen magnum), spherical shell or boundary element models provide only approximate electrical propagation from cortex to the surface, and more detailed (FDM or FEM) volumetric models are needed. Finally, the position of the electrodes must be specified accurately, for example, with geodesic photogrammetry [4].

By describing the positions of electrodes and cortical surface targets, an accurate electrical head model also supports dense array approaches to transcranial current injection. The electrical head model can be validated with bounded Electrical Impedance Tomography (bEIT), in which current injection and potential recovery are analyzed, within the bounds of the tissue geometry from MRI, to test whether the electrical conductivity of head tissues estimated by the model is accurate in predicting the recovered potentials [5, 6]. With an accurate electrical head model, it is possible to modulate brain activity noninvasively, using dense array transcranial Direct Current Stimulation (tDCS), or transcranial Alternating Current Stimulation (tACS), in which patterns of source and sink electrodes are computationally optimized to target specific cerebral sites [7, 8].

In the present report, we review the methods implemented in BrainK. In addition, we report validation studies for accuracy and efficiency of both tissue segmentation and cortical surface extraction. Klauschen et al. [9] evaluated automatic tools for head tissue segmentation from MR images. These included FSL [10], FreeSurfer [11, 12], and SPM [13]. Although BrainK is designed as an automated tool, visualization and editing capability is provided to allow adaptation to unique image properties, such as the presence of lesions or tissue anomalies.

## 2. Overview of BrainK

**Figure 1:**
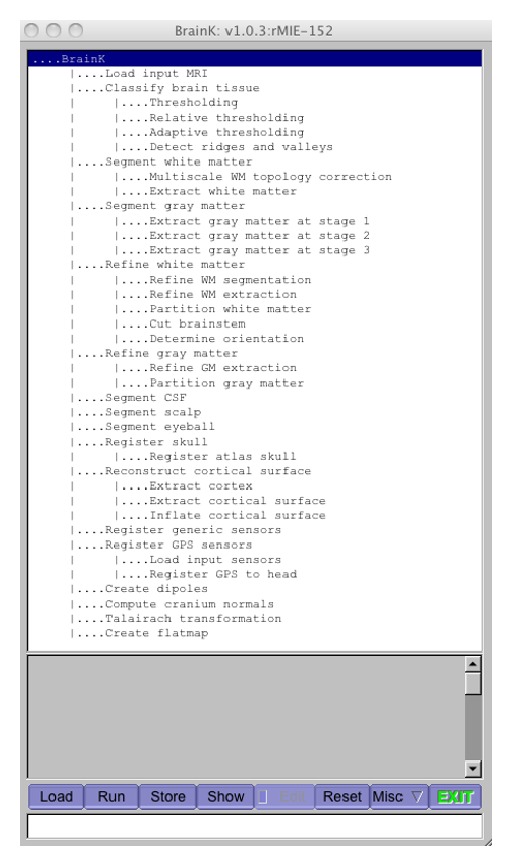
BrainK GUI.

**Figure 2:**
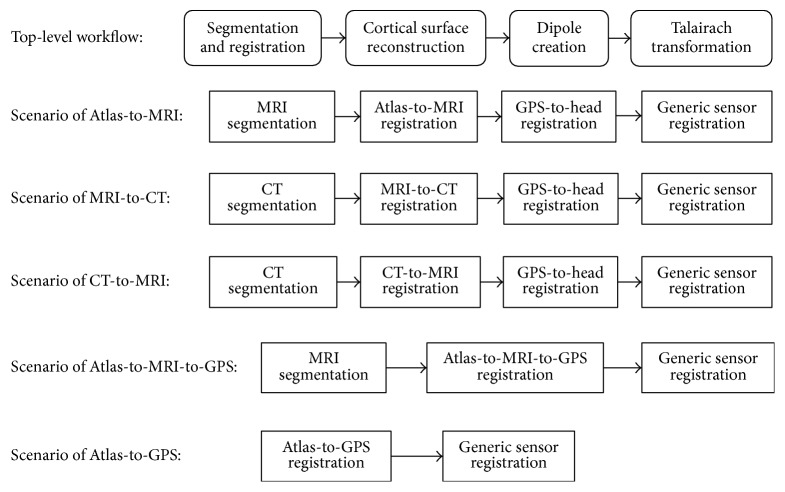
BrainK architecture.

Figure 1 shows the graphical user interface for BrainK, including the multiple steps of image processing. Although maximal accuracy for EEG source localization or transcranial neuromodulation requires the full complement of MRI, CT, and sensor photogrammetry, it is important to optimize the results possible with the imaging data available for a particular subject or patient.  Figure 2 shows the architecture of BrainK that incorporates specific workflows designed to adapt to the available data for the person. Although it is possible to assume standard sensor positions for BrainK (when sensor placement is referenced to skull fiducials, as with the Geodesic Sensor Net), a more accurate EEG source localization workflow begins with sensor positions from photogrammetry, such as what is provided by the Geodesic Photogrammetry System (GPS). If no imaging data is available for the person, an Atlas head model, constructed from a database of MRIs and CTs for the appropriate ages [14], is then warped with nonlinear registration to fit the sensor positions (and thus the person's head shape), producing a* conformal Atlas head model*. If only a structural MRI (typically a volumetric T1) is available for the person, the sensor positions are registered to the MRI, a database skull (from CT) is registered to the MRI, and tissue segmentation and cortical surface extraction are conducted to create the* individual head model with Atlas skull*. The skull compartment includes either the original CT Hounsfield units for estimating bone density (and thus conductivity) voxel by voxel or a single skull segment, depending on the FDM computational model that will use BrainK's head model output. If both the CT and MRI are available for the person, the MRI is registered to the CT, which has more accurate dimensions than the MRI, to create the* individual head model with individual CT skull.*

For all workflows when the T1 MRI is available for the person, BrainK segmentation identifies the white matter (WM) and the gray matter (GM). It partitions these into two hemispheres and it differentiates cerebellum from cerebrum. In addition, an entire head mask and the two eyeballs are separated as well in the segmentation component. The eyeballs may be important for the electrical head model because of the large far fields generated by their cornea-retinal potentials (that must be separated from the brain signal in the EEG). For all workflow scenarios, a generic spherical sensor cloud is warped onto the head contour of the subject. For the scenarios of Atlas-to-MRI, MRI-to-CT, and CT-to-MRI, an additional GPS-to-head registration procedure is conducted to register the specific GPS sensor cloud, from one individual Geodesic Sensor Net application, onto the head contour. For the scenarios of Atlas-to-MRI-to-GPS and Atlas-to-GPS, the individual GPS data has already played a role in the skull registration and has been aligned with the head contour. In all scenarios, the skull registration is conducted, such that the resulting head segmentation includes the following tissue types: WM, GM, CSF, bone, flesh, and eyeball, in which the WM and the GM are further partitioned into two cerebral hemispheres and the cerebellum.

## 3. Methods in BrainK

### 3.1. Image Segmentation

The MRI segmentation implements a cascade of automatic segmentation procedures in order to identify and separate the head tissues required for electrical head modeling, including the scalp, skull, cerebrospinal fluid (CSF), brain (gray and white matter), and the eyeballs. Finally, cortical surface extraction is performed to allow characterization of the normal of the cortical surface (allowing dipoles to be fit perpendicular to the cortex). The cascade mainly consists of two types of procedures: voxel classification on a region of interest (ROI) and the extraction of certain anatomical structures. The classification procedure aims at labeling all voxels in the ROI into different tissue types. The extraction procedure performs morphological operations on the voxel classification so that a certain anatomical structure, such as the WM, the GM, or the scalp, is separated from erroneous (false positive) segmentation results.

**Figure 3:**
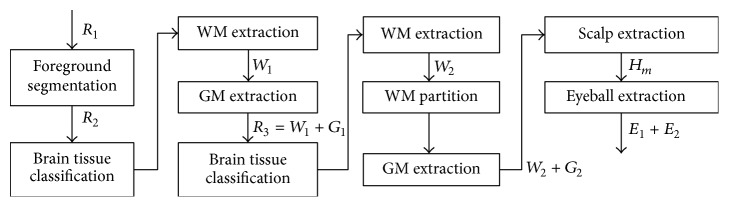
MRI segmentation workflow.

**Figure 4:**
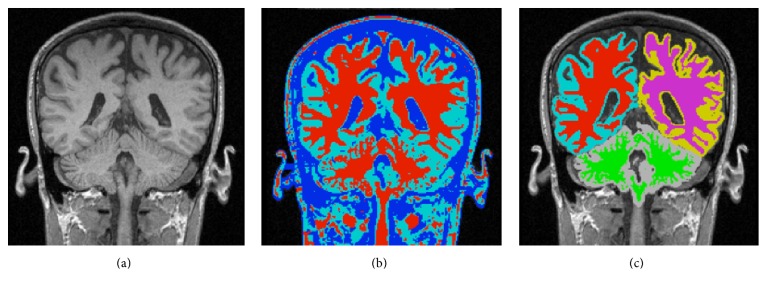
MRI segmentation. (a) MR image. (b) RT result on the foreground. (c) MRI segmentation result.

Given the MRI data, the segmentation cascade as shown in Figure 3 first takes the entire MR image space as the first ROI, *R*_1_, and classifies all the voxels into two types: foreground and background. The following foreground extraction procedure does simple morphological operations to further improve the foreground segmentation resulting in the second ROI, *R*_2_, in which all voxels are classified into three tissue types: WM, GM, and CSF, as shown in Figure 4(b). The major operation performed in this procedure is BrainK's novel relative thresholding (RT) technique (Section 3.1.1) [15]. Given the voxel classification on *R*_2_, the WM extraction procedure then produces an initial WM volume *W*_1_, which is used as the basis to identify the initial GM volume *G*_1_ by the GM extraction procedure. Next, the union of *G*_1_ and *W*_1_ is taken as the third ROI, *R*_3_, in which the WM/GM classification is refined with a different RT scheme. The new WM volume is then processed by the WM extraction procedure and a WM partition procedure, ending up with the second WM volume *W*_2_.

This WM partition procedure not only separates the two cerebral hemispheres from each other but also makes an optimal cut between the cerebral WM and the cerebellar WM with the well-known maximum flow algorithm [16]. The result is specification of the following tissue types in *W*_2_: two types of cerebral WM for the two hemispheres, respectively, and the cerebellar WM. *W*_2_ then forms the basis for extracting the new GM volume *G*_2_, which includes the two types of cerebral GM, respectively, for the two hemispheres and the cerebellar GM, as shown in Figure 4(c). The brain tissue segmentation (*W*_2_ plus *G*_2_) is then taken as a reference data for the scalp segmentation resulting in the head mask *H*_*m*_. The scalp segmentation then conducts a binary thresholding procedure on *R*_1_.

To achieve the above segmentation steps, BrainK implements a novel morphological image analysis (SMIA) technique (Section 3.1.3) and a cell complex based morphometric image analysis (CCMIA) method (Section 3.1.4). These play key roles in the WM extraction, topology correction, GM extraction, and scalp extraction. Together with the relative thresholding RT method, the SMIA and CCMIA steps form the core techniques used in BrainK and will be described in the following subsections in detail. The eyeball extraction from the head mask is a special procedure using a priori knowledge about the eyeballs.

Although segmentation of the skull from the MRI is an important challenge, the characterization of bone properties is poor in the typical T1 MRI sequence compared to that provided by CT. The Hounsfield units provided by the CT image (measuring X-ray attenuation) are directly proportional to bone density and thus provide important information on the relative tissue properties of trabecular skull. For example, the CT Hounsfield units correlate *r* = 0.83 with water content in trabecular skull [17].

The CT image segmentation procedure first performs the simple yet robust thresholding [18] so that the entire image is classified into three voxel types: bone, flesh, and background. The original Hounsfield units are then retained as bone density and conductivity estimates. Morphology operations follow to smooth the initial segmentation. Finally, the maximum flow algorithm is applied to separate the brain volume from the rest of the soft tissue so that the cut between them is minimized. CT segmentation results in a voxel classification as well as a brain volume wrapped up by the cranium and eventually there are four tissue types in the segmentation: bone, flesh, brain, and background.

#### 3.1.1. Relative Thresholding (RT)

A well-known and prominent image artifact that challenges MR image segmentation is the intensity inhomogeneity (IIH) due to spatial distortions in the radio frequency (RF) gain in the RF coil [19]. The presence of IIH leads to a shading effect over the image and to significant overlap between histograms of different tissues. The result is that intensity based methods, such as thresholding and clustering, are typically unreliable in brain tissue segmentation. IIH correction can be performed prior to image segmentation, but the procedure may eliminate not only the image artifacts but also image signals that provide important information. When the correction is performed together with the segmentation, additional degrees of freedom are introduced into the problem formulation, making the optimization procedure more susceptible to local optima. In this paper, we present a new segmentation method referred to as relative thresholding (RT), which uses two global relative thresholds to compare with the local intensity contrasts, thereby bringing global and local information to segment the ROI into WM, GM, and CSF.

The nature of RT makes it robust against the influence of IIT without introducing an explicit IIH correction. Like traditional thresholding methods, RT enables exhaustive searching without possibilities of being trapped into local optima. It also incorporates various a priori knowledge such that it is robust to intersubject variability and to the convolution of cortical structures. Finally, RT conducts brain tissue segmentation without the need of a prior step of brain volume extraction.

In spite of intersubject variability, complexity of cortical structures, and variability on MR imaging sequences, we can make the following a priori structural, geometrical, and radiological observations: (1) Skull, CSF, GM, and WM form a layered structure from outside to inside; (2) the average intensities of skull, CSF, GM, and WM in local regions are in ascending order in T1-weighted MRI; (3) the cortex thickness is nearly uniform and is a very small value compared to the size of brains; in addition to the structural modeling described above, we also formulate an image model by incorporating a multiplicative low-frequency bias field *b*_*i*_ and an additive noise field *ρ*_*i*_ as follows:

(1) $$\begin{array}{c} y_{i}=b_{i}y_{i}^{\prime }+\rho_{i}, \end{array}$$

where *y*_*i*_ is the observed intensity at the *i*th voxel and *y*_*i*_′ is the unobservable true intensity without the influences of IIH and noise.

The structural modeling and the image modeling enable two procedures, both of which compare the intensity of a subject voxel *x*_*i*_ with that of a reference voxel *x*_*j*_ on its gradient path, which is a set of ordered voxels emanating from *x*_*i*_ and following the gradient direction at each voxel in the path. The first procedure, GM-WM differentiation, scans all voxels in the ROI, which are initialized as WM, and labels them as GM if there is such a reference voxel *x*_*j*_ that its distance from *x*_*i*_ is less than a distance threshold *d*_*gw*_ and the ratio between their smoothed intensities *z*_*i*_/*z*_*j*_ is less than a relative threshold *t*_*gw*_; the second procedure, CSF-GM differentiation, scans each GM voxel *x*_*i*_ and relabels it as CSF if there is such a WM voxel *x*_*k*_ on its gradient path whose distance from *x*_*i*_ is less than another distance threshold *d*_*cw*_  (*d*_*cw*_ > *d*_*gw*_) and the ratio between their smoothed intensities *z*_*i*_/*z*_*k*_ is less than another relative threshold *t*_*cw*_  (*t*_*cw*_ < *t*_*gw*_).

In order to find the optimal relative thresholds, BrainK exhaustively tries each pair among the Cartesian product of the candidate GM/WM relative threshold set and the candidate CSF/WM relative threshold set and chooses the pair that minimizes an objective function *h* = *w*_*w*_ *h*_*w*_ + *w*_*g*_ *h*_*g*_ + *w*_*c*_ *h*_*c*_. In this equation, *h*_*w*_ is a metric measuring the homogeneity of the WM object and formulated as the sum of smoothed intensity differences of any pair of adjacent voxels labeled as WM. *h*_*g*_ and *h*_*c*_ are formulated in the same spirit for GM and CSF, respectively. *w*_*w*_, *w*_*g*_, and *w*_*c*_ are three weighting coefficients.

The second RT scheme takes the optimal relative thresholds and differs from the first scheme on the question of how to determine the reference intensity compared to the intensity of the subject voxel. The ROI *R*_3_ is first reset and then an initial set *W* ^0^ of WM voxels are determined by thresholding *R*_3_ with a traditional threshold that maximizes the objective function *f* = *f*_*e*_ − *f*_*r*_, where *f*_*e*_ is the sum of intensity differences of all *m* pairs of adjacent voxels labeled as WM and non-WM and *f*_*r*_ is the sum of differences of the *m* pairs of adjacent voxels labeled as WM and with the greatest intensity differences. A minimal cardinality of *W* ^0^ is set to improve the robustness. The remaining region of *R*_3_ is then segmented as a procedure of iteratively dilating *W* ^0^ by using the optimal GM/WM relative threshold to compare the subject voxel's intensity to a reference intensity computed by considering the WM voxels near the subject voxel.

#### 3.1.2. GM and WM Extraction

The GM and WM extraction procedures are responsible for extracting the GM and the WM structure from the raw voxel classification in the ROI. We describe GM extraction first, assuming that WM extraction is already done. The GM extraction utilizes the following a priori knowledge: (1) the thickness of cortex is nearly uniform; (2) GM wraps around WM such that two tissues form a layered structure; (3) the average gray level of GM is lower than that of WM in any local region. Given the extracted WM volume *W*_2_, the GM volume is first created by a gradient flow process, in which any voxel originally labeled as GM in the ROI is taken as the true GM if it can reach any WM voxels by following the gradient path emanating from itself within a given distance threshold. Each step in the gradient path is determined by looking at the gradient direction for each voxel. This initial GM volume is then further processed by traditional morphological operations to improve the segmentation, ending up with the GM volume *G*_2_.

WM extraction is a more challenging process than GM extraction because it is responsible for extraction of WM itself, and it is also responsible for the brain volume extraction. The following a priori knowledge forms the basis of WM extraction: the WM is highly connected, but it has no topological defects. Topological defects or false positives present narrow bottlenecks. The extraction mainly consists of two steps: (1) WM localization, in which the center of one of the hemispheres is determined; (2) separation of WM from false positives. The localization of the WM center is essentially finding the WM voxel with the highest connectivity, which means that some quantitative measurement of connectivity should be taken for all WM voxels. Since topological defects, such as a tunnel inside the WM, can greatly influence the geometric measurement of the connectivity, the WM should be first topology-corrected before the connectivity measurement. Because the cortex surface (ribbon) must be defined in relation to the correct topology of the WM, WM topology correction is also essential for valid cortical surface reconstruction. BrainK introduces a skeletonization based morphological image analysis (SMIA) method and a cell complex based morphometric image analysis (CCMIA) framework. The following sections describe how these methods play central roles in WM extraction through achieving accurate connectivity measurement and topology correction.

#### 3.1.3. Skeletonization Based Morphological Image Analysis (SMIA)

BrainK's SMIA framework consists of a* surface skeletonization* procedure and a* curve skeletonization* procedure, both of which are based on its extensive topological point classification. Given a binary image, we say its foreground *F* has a* handle* whenever there is a closed path in *F* that cannot be deformed through connected deformations in *F* to a single point. A handle in *F* is referred to as a* tunnel* in its complement $\overset{-}{F}$. A point in *F* is simple if it can be added to or removed from *F* without changing the topology of both *F* and $\overset{-}{F}$. A* simple point*[20] is a central concept in digital topology and is the basis of our definition of what we call a* thick-simple point*. A point *P* is thick-simple with respect to *F* if it is simple with respect to *F* and the removal of *P* and any of its neighbors from *F* does not increase the number of tunnels and number of connected components in *F*. A point is* thin-simple* if it is simple but not thick-simple and can be classified as thick-surface and thick-curve points. Thick-simple points can also be further classified as surface-edge, curve-end, and other point types.

There are two steps for BrainK's surface skeletonization based on the above point classification:* thick-surface skeletonization* and* thin-surface skeletonization*. The former results in a discrete surface with thickness of at most two voxels, and the latter results in a final thin surface skeleton of one-voxel thickness. The thick-surface skeletonization iteratively removes boundary points of the object that are thick-simple at the moment of removal. The thin-surface skeletonization iteratively removes boundary points of the thick-surface skeleton that are thick-surface points, thick-surface-edge points, or thick curve-end points at the moment of removal. The curve skeletonization procedure iteratively removes the boundary points of the object that are thick-curve points or thick-simple, but not curve-end points, at the moment of removal. As a side product, BrainK's surface skeletonization procedure gives each skeleton point a depth metric as the distance from the point to the boundary, whereas the curve skeleton procedure gives each skeleton point a wideness metric.

If the curve skeletonization procedure is conducted at a certain scale, then the points that become the thin-curve points can be checked if they are on a handle of the object. Removal of such handle points then corrects the topology defects by cutting the handle. BrainK supports a multiscale topology correction [21] method on the WM object so that the WM extraction is made more robust, and then the topology-corrected cortex can be generated based on the WM.

#### 3.1.4. Cell Complex Based Morphometric Image Analysis (CCMIA)

CCMIA is motivated by the goal of representing the true connectivity of an object's skeleton considering the convolution of such objects as the cerebral WM, where neither the depth nor the wideness of the structure is good enough for this need. It is essentially a series of transformations on a space called the* cell complex* [22]. A cell complex is a topological space composed of points, segments, polygons, polyhedrons, and the generation to polytope in any dimension. Given a set of voxels *X* in the 3D binary image, we can construct a 3-dimensional cell complex, that is,* 3-complex*, by creating a point for each voxel, an edge for every two connected points, a triangle for every three interconnected edges, and a tetrahedron for every four interconnected triangles. CCMIA proceeds as transforming the 3-complex to a 2-complex with points, segments, and polygons only and then the 2-complex to a 1-complex with points and segments only. The 3-2 transformation iteratively removes those boundary tetrahedrons by dropping one face *f* while a connectivity metric and a depth metric are accumulated on each *f*′ of the remaining three faces for the removed tetrahedron as depth(*f*′) = depth(*f*) + *d*(*f*, *f*′), while *d*  represents the distance between the centers of the two faces. In a similar spirit, the 2-complex can be transformed to 1-complex and the remaining edges can be set with two accumulated metrics as wideness(*e*′) = max(widness(*e*′), wideness(*e*) + *d*(*e*, *e*′)) and connectivity(*e*′) = connectivity(*e*′) + connectivity(*e*) + *d*(*e*, *e*′).

After the raw WM object is topologically corrected, its surface skeleton is processed by CCMIA ending up with the 1-complex with points and edges only. The point incident to the edge of the greatest connectivity is identified as the WM center and a wideness threshold is used to break the bottleneck between the true WM and the false positives. Finally, the WM surface skeleton is dilated to restore the volumetric object.

### 3.2. Registration

Given the varying data available, flexible and accurate registration is an essential BrainK function. Whereas the MR image accurately differentiates soft tissues, the CT image accurately represents bone. When individual CT or even MRI is not available, BrainK provides registration of the sensor photogrammetry data to a digital head tissue Atlas of the appropriate age and gender. BrainK integrates both soft tissues and bones from multimodal images and from existing digital Atlases by various image registration techniques.

Image registration is a problem of finding optimal geometric transformations between images so that each point of one image can be mapped to a corresponding point of another image. There are generally rigid transformations and nonrigid ones. Rigid transformation involves only translation and rotation. Affine transformation is a typical nonrigid transformation and allows scaling and shearing. Another form of nonrigid transformation is an elastic transformation, allowing local deformation based on models from elasticity theory. BrainK implements rigid and affine transformations, as well as a landmark-based elastic transformation using thin-plate spline theory [23]. For different registration purposes, either one or more of them is used so that the simple transformation serves as initial state for the more sophisticated transformation.

Both the rigid transformation and the affine transformation can be represented by a simple 3 × 4 matrix. The key issue in applying such transformations is to optimize the similarity measure between the source and the target image, which in BrainK is represented in terms of landmarks. For the thin-plate spline transformation, the displacement field at any point is computed by interpolation from the vectors defined by a set of source landmarks and a set of target landmarks. It can be seen that landmark extraction plays a central role in all three transformations. BrainK is optimized for extracting landmarks based on the preceding image segmentation and on a priori anatomical knowledge. All registration procedures in BrainK undergo three steps: (1) landmarks extraction, (2) transformation coefficients determination, and (3) transformation execution. Note that three transformations all use landmarks but in different ways. The landmarks in rigid and affine transformation are used for constructing the similarity measurement, while there are source landmarks and target landmarks in thin-plate spine transformations and they are mapped to build the displacement field.

The thin-plate spline theory is based on an analogy to the approximate shape of thin metal plates deflected by normal forces at discrete points. Given *n* source landmarks *P*_*i*_ = (*x*_*i*_, *y*_*i*_, *z*_*i*_) in the source image and the corresponding target landmarks *P*_*i*_′ = (*x*_*i*_′, *y*_*i*_′, *z*_*i*_′) in the target image, the thin-plate spline transformation maps any point *P* = (*x*, *y*, *z*) in the source image to the point *P*′ = (*x*′, *y*′, *z*′) in the target image as follows:

(2) x′=a0+a1x+a2y+a3z+∑i=1ndiri2ln⁡ri2,y′=b0+b1x+b2y+b3z+∑i=1neiri2ln⁡ri2,z′=c0+c1x+c2y+c3z+∑i=1nfiri2ln⁡ri2,

where *r*_*i*_^2^ = (*x* − *x*_*i*_)^2^ + (*y* − *y*_*i*_)^2^ + (*z* − *z*_*i*_)^2^. The coefficients in the above equations together form a (*n* + 4) × 3 matrix *W*, which can be obtained by solving the equation *LW* = *M* that forces the matching of *P*_*i*_ and *P*_*i*_′, where *M* is formed by organizing the coordinates of *P*_*i*_′ = (*x*_*i*_′, *y*_*i*_′, *z*_*i*_′) and *L* is formed by composing the coordinates *P*_*i*_ = (*x*_*i*_, *y*_*i*_, *z*_*i*_) and the distance information *r*_*i*_^2^ln⁡*r*_*i*_^2^.

When the individual CT as well as the MRI data is available for the person, the registration component in the MRI-to-CT scenario first segments the CT into the following tissue types: bone, brain, flesh, and background. The MRI segmentation is then aligned with the CT segmentation so that the brain tissues and the eyeballs recognized in the MRI data are properly transformed and put into the brain region defined by the CT data. The result of this transformation is that the entire head segmentation (WM, GM, CSF, bone, eyeball, and flesh) is now registered with the CT volume, where the CSF is redefined as the “brain” region recognized in the CT segmentation, but now subtracting the WM and GM that were inserted from the MRI segmentation.

When both MRI and CT are available, the user could also choose the CT-to-MRI registration so that the bone recognized in the CT is transformed and put into the MRI segmentation wrapping around the brain and the CSF is inserted between the brain and the cranium. However, the MRI-to-CT registration is preferred when the CT covers the entire head of the subject because MRI imaging introduces more geometry distortion than CT. However, when the CT only covers the cranium region and the MRI data includes the face and the jaw, the user could consider the CT-to-MRI registration in order to have adequate face and jaw data for the electrical head model.

When only the MRI data is available for the given subject, BrainK supports the skull Atlas-to-MRI registration, in which the bone tissue from an Atlas dataset is warped into the MRI space and the CSF is also taken as those regions between the cranium and the brain. Any region within the head mask that is not occupied by the GM, the WM, the CSF, the eyeballs, or the bone is set to* flesh*.

When only the MRI and the individual GPS data are available and if the MRI is badly warped (dimensionally distorted), the user could choose the MRI-to-GPS registration, so that the head region is warped to match the GPS sensor cloud, which has good metric properties of the actual head shape. The result is transformed segmentation data, which can be further processed by the Atlas-to-MRI registration described above.

Thus, the skull registration can be adapted to fit the available image data for the individual. When only the individual GPS data is available for a given subject, an Atlas dataset with all required tissue types can be warped to match the GPS sensor cloud with the so-called Atlas-to-GPS registration, producing an individual conformal Atlas that has the appropriate shape of the volume conductor for electrical head modeling.

#### 3.2.1. MRI/CT Registration

After MRI segmentation, we already have the MRI brain volume that is composed of WM and GM, but not CSF. The CT brain volume occupies spaces of WM, GM, and CSF. By applying morphological closing at a proper scale on the union of the MRI WM and GM, we obtain the MRI brain mask *B*_*m*_, which has some inner CSF space filled and the brain contour smoothed but still misses some CSF voxels mainly wrapping around the superior contour of the brain. MRI segmentation also produces the MRI head mask *H*_*m*_ and the two eyeball masks *E*_1_ and *E*_2_. Correspondingly, we have the CT brain mask *B*_*c*_ and the CT head mask *H*_*c*_. These data form the input set used to determine the transformation coefficients of the MRI/CT registration.

MRI/CT registration undergoes a rigid transformation followed by an affine transformation. Different sets of landmarks are extracted for the two transformations. The rigid transformation aims at only providing a good initial state for the affine transformation and its landmarks are simply those bordering voxels around *B*_*m*_. The similarity measure to optimize the rigid transformation is the sum of squares of the distances from all landmarks to the contour of *B*_*c*_. The optimal coefficients are found by multiscale exhaustive searching with acceptable computational efficiency and good global optimization performance.

The landmarks for the affine transformation are more sophisticated and consist of two parts: the inferior landmarks and the superior landmarks. The inferior landmarks are from the border voxels of the inferior part of *B*_*m*_ while the superior landmarks are from the superior contour of *H*_*m*_. The inferior and the superior landmarks are partitioned so that they “visually” wrap up *B*_*m*_ but do not overlap. The motivation of using two sets of landmarks is to align MRI with CT in terms of both the brain matching and the scalp matching. However, the brain volume in the CT may contain significantly more CSF than that in the MRI at the superior part of the volume and we make the observation that their contours have reliable matching only at the inferior part of the brain. The landmark extraction thus essentially involves the partitioning of the cephalic space into the inferior part and the superior part. BrainK takes the following three landmarks as reference points to partition the cephalic space: the center of the brain mask *B*_*m*_ and the centers of the two eyeball masks *E*_1_ and *E*_2_. All three points can be automatically determined.

**Figure 5:**
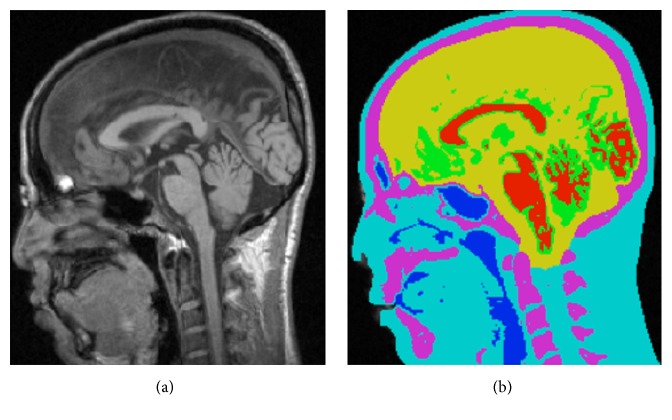
MRI-to-CT registration. (a) Transformed MRI. (b) Fusion of MRI and CT segmentation.

The similarity measure for the affine transformation is formulated as the sum of two terms. One is the sum of squares of the distances from all inferior landmarks to the contour of *B*_*c*_ and the other is the sum of squares of the distances from all superior landmarks to the contour of *H*_*c*_. A gradient-descent optimization method is used to obtain the optimal affine coefficients. When all the optimal coefficients of both the rigid and the affine transformation are obtained, the MRI/CT registration can be performed from one direction while the other can be achieved by simply inverting the coefficient matrix. The result of MRI-to-CT registration is shown in Figure 5.

#### 3.2.2. Skull Atlas-to-MRI Registration

**Figure 6:**
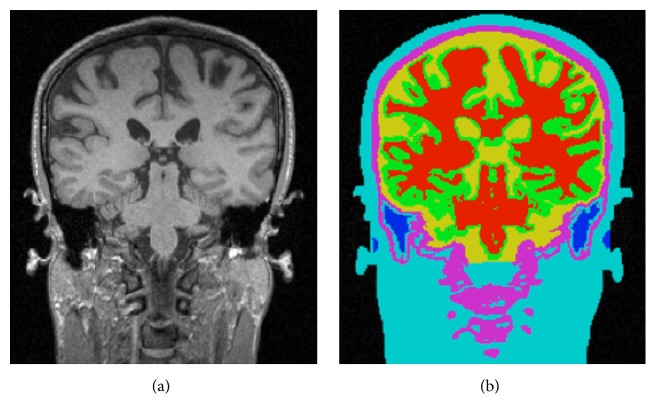
Skull Atlas-to-MRI registration. (a) Transformed MRI. (b) Fusion of Atlas skull and MRI segmentation.

Since the Atlas and the individual subject can have significant geometric differences, the Atlas-to-MRI registration undergoes an elastic thin-plate spline transformation following the rigid and the affine transformations. The rigid transformation provides a good initial state for the affine transformation, which in turn provides a good initial state for the elastic local transformation. The result of the Atlas-to-MRI registration is illustrated in Figure 6.

The rigid transformation and the affine transformation use the same set of landmarks to formulate the similarity measures. Let *B*_*m*_ be the MRI brain mask defined as in MRI/CT registration. Let *B*_*a*_ be the Atlas brain mask defined in the same way as *B*_*m*_. The landmarks are then the set of border voxels of *B*_*m*_ and the similarity measure is the sum of squares of distances from all landmarks to the contour of *B*_*a*_. The coefficients of the rigid transformation are found by multiscale exhaustive searching while those of the affine transformation are determined by gradient-descent searching.

The source landmarks used in the thin-plate spline transformation include several parts. The two primary parts are the inferior source landmarks and the superior source landmarks. Inferior source landmarks are distributed over the inferior part of the contour of the Atlas brain mask *B*_*a*_. The superior source landmarks are placed over the superior part of the contour of the Atlas head mask *H*_*a*_. Their extraction is very similar to the extraction of those used in the MRI/CT registration, but they are much more sparsely distributed to reduce the computational overhead.

The corresponding target landmarks in the subject head are determined automatically after the rigid and affine transformation are performed. For each superior source landmark, the corresponding superior target landmark is obtained as the intersection of the contour of the subject head mask *H*_*m*_ and the line between the superior source landmark and the subject brain center. We have two ways to obtain the inferior target landmarks according to their locations. The first method is the same as that obtaining the superior target landmarks and works for the posterior part of the inferior landmarks that are distributed around the cerebellum. For the second method that works for the anterior part of the inferior landmarks, we compute a field of distance from the contour of the brain mask *B*_*m*_, and each inferior target landmark, which should be on the contour of *B*_*m*_, is obtained by tracing from the corresponding source landmark following the gradient of the distance field.

In the procedure of the registration, the Atlas skull's thickness is adjusted according to an estimated ratio of the subject skull's thickness to the Atlas skull's thickness. This is achieved by using another set of source landmarks and another set of target landmarks. For each superior source landmark, we determine its “mate landmark” over the inner side of the Atlas skull and on the line between the superior source landmark and the Atlas brain center. For each superior target landmark, there is also a “mate landmark” on the line between the superior target landmark and the subject brain center. For each pair of the superior source landmark *S*_*i*_ and its mate landmark *S*_*i*_′, we have a distance *d*_*i*_^*S*^; for each pair of the superior target landmark *T*_*i*_ and its mate landmark *T*_*i*_′, we have a distance *d*_*i*_^*T*^. A global ratio is then defined as *d*_*i*_^*T*^/*d*_*i*_^*S*^ and its optimum is determined as the highest value so that the majority (such as 90%) of the superior target mate landmarks are off the mask *B*_*m*_. For the few superior target mate landmarks that are within *B*_*m*_ according to the global ratio, the global value is decreased locally so that they are off the mask *B*_*m*_.

For each posterior inferior source landmark, we determine its mate landmark over the outside of the Atlas skull and on the line between the source landmark and the Atlas brain center. For each posterior inferior target landmark, we also have a mate landmark on the line between the target landmark and the subject brain center while its location is determined according to the global thickness ratio described above. In order to further improve alignment performance, there are few other landmarks such as the centers of the eyeballs. We have a specific algorithm to automatically detect the subject's eyeballs, but the details are not described here for the limit of space. When all source landmarks and all target landmarks are determined, the transformation for any point in the space can be calculated as shown in (2).

#### 3.2.3. Registrations to the EEG Sensor Positions

**Figure 7:**
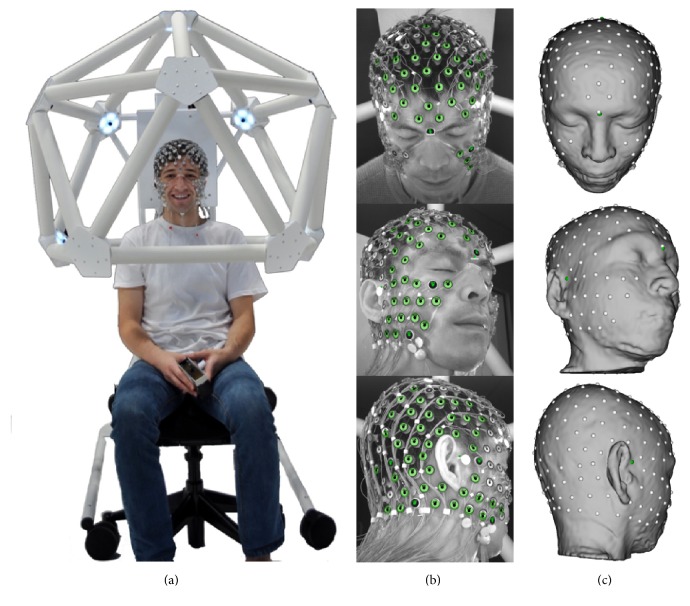
(a) Geodesic Photogrammetry System (GPS). (b) Identification of EEG sensors in the GPS software. (c) Registration of EEG sensors with the MRI head surface in BrainK.

The EEG sensor positions are automatically localized in 3D with the Geodesic Photogrammetry System (GPS). GPS captures images from 11 cameras in fixed positions to allow identification of the sensors in the images and then computation of the 3D coordinates (Figure 7). The Atlas-to-MRI-to-GPS registration then consists of two stages, the MRI-to-GPS registration and then the Atlas-to-MRI registration, using the newly transformed MRI. Atlas-to-MRI registration has been described in the above subsection, and this subsection only deals with the MRI-to-GPS registration. There are three steps. The first can be an automatic rigid transformation with other alternatives described below. The second is an affine transformation. The first two can bring the MRI to a good alignment state with the GPS data, but the GPS points may still be off the scalp. The third step then performs thin-plate spline transformation to deform the MRI so that each GPS point locates on the scalp. The landmarks used in the first two steps are the GPS points themselves, and the similarity measure is the sum of squares of the distances from the landmarks to the MRI scalp, that is, the contour of the head mask *H*_*m*_. For the thin-plate spline transformation, the target landmarks are the GPS points while the source landmarks are those points on the contour of *H*_*m*_ so that each one is on the line connecting the brain center and the corresponding target landmark.

The first automatic step of the MRI-to-GPS registration can be replaced by a transformation with the user providing three fiducial landmarks on the MRI space: the nasion and the left and right periauricular points (where the jaw hinge meets the skull). BrainK is able to stretch the MRI so that the user-specified landmarks match the corresponding ones in the GPS space. This initial state can be directly fed to the thin-plate spline transformation or it may pass through an intermediate affine transformation. BrainK also allows the user to specify only the nasion landmark and does rigid transformation in the first step while keeping the corresponding nasion landmarks matched.

The Atlas-to-GPS registration is very similar to the MRI-to-GPS registration, except that the Atlas typically has greater geometric differences from the GPS than the subject's own MRI. The steps are similar to those for the MRI-to-GPS transformation just described. For the MRI-to-GPS registration, however, if they are to be used, the fiducial landmarks have to be manually marked on the MRI, whereas the fiducials are included in the digital Atlas dataset for the Atlas-to-GPS registration. In addition, one more fiducial landmark, the vertex, is used to accommodate the greater geometric difference between the Atlas and the subject.

The GPS-to-head registration can be seen as the inverse of the registration of MRI-to-GPS, but no thin-plate spline transformation is needed. The initial transformation, either automatic rigid transformation or the stretching in terms of fiducial landmarks matching, can put the GPS in a good initial state. If needed, an affine transformation can be performed to further improve the alignment. The final adjustment then moves each GPS point onto the scalp, if it is not on it yet, with an end result that is similar to finding the corresponding landmarks in the MRI-to-GPS registration.

When GPS is not available, a set of generic sensor positions may be used, such as those from the appropriate Geodesic Sensor Net (channel count and size) selected from the database. This registration has two steps. First, the generic spherical sensor cloud undergoes a translation transformation so that the center of the sensor point cloud overlaps with the center of the brain. Second, in a procedure that matches the physical conformation of the geodesic tension structure to the head, each sensor is mapped to a point on the scalp by the ray emanating from the brain center to the sensor's position.

### 3.3. Cortical Surface Reconstruction and Inflation

The well-known marching-cube isosurface algorithm [24] is performed on the cerebral cortex volume of each hemisphere to reconstruct the cortical surface mesh. In addition, BrainK supports cortical surface inflation by iteratively updating all points in the mesh, such that the new position of each point is the weighted sum of its old values with the weighted sum of the centroids of the triangles that are incident to the point.

### 3.4. Dipole Tessellation

The dipole tessellation operates in two modes. If the individual's MRI is available, the cortical surface is extracted and tessellated into patches, and a dipole is fit to each patch. If it is not available, then dipole* triples* (fitting *x*, *y*, *z* components of the unknown dipole orientation) are distributed evenly through the cortical gray matter.

Whereas a trivial algorithm can distribute the triples, the even distribution of the oriented dipoles over the cortical surface is formulated as a sophisticated* graph partition* [25] problem. Given the cortical mesh from marching cubes, composed of a great number of interconnected tiny triangles, a graph is first constructed so that each* vertex* corresponds to a triangle and each* edge* incident to two vertices corresponds to the adjacency between the two corresponding triangles. The graph partition algorithm then divides the entire graph into a set of parts, with the constraints that (1) the mesh is divided into the same number of patches as the desired number of oriented dipoles, (2) all patches are very compact (the more like to a square or circle, the better), and (3) all patches have similar areas. The graph partition library Chaco [26] is used for the implementation.

**Figure 8:**
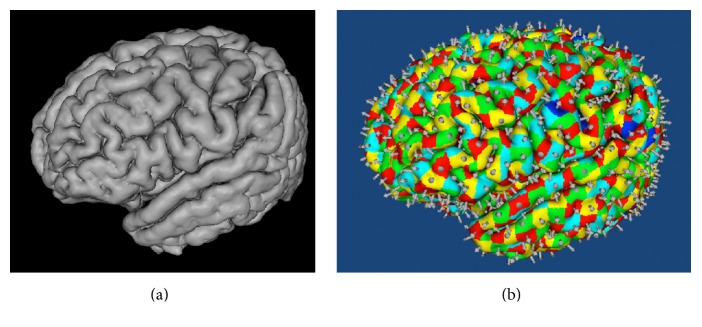
Cortical surface (a) and tessellation of the surface into ~1 cm^2^ patches (about 1200 per hemisphere), each with an oriented equivalent dipole (b). Color is used here only to separate the cortical patches, such that every patch has a different color from its neighbors.

Given the mesh partition performed as above, an equivalent oriented dipole is then defined for each patch, generated at the center of the patch, with its orientation defined by the mean of all surface normals of the triangles in the patch, weighted by their areas. This equivalent dipole thus reflects the orientation of the electrical source if the entire patch was synchronously active, thereby serving as a useful approximation for the resolution of cortical activity that is set by the graph partitioning decision. The cortical surface and the oriented dipoles are displayed in Figure 8.

### 3.5. Talairach Transformation

The Talairach transformation component brings all the data, the segmentation, the cortical surfaces, the sensors, and the dipoles, into the standard Talairach space [27, 28]. The transformation is determined with three points specified by the user: the anterior commissure (AC), the posterior commissure (PC), and one point forming the middle plane with AC and PC. The procedure maintains the topology correctness of the cortical surface that is guaranteed in either the MRI segmentation component or the Atlas dataset and then maintained through each registration operation.

## 4. Evaluation of BrainK's Tissue Segmentation and Cortical Surface Extraction

BrainK provides certain algorithms, such as skull bone density (X-ray CT) image registration and cortical surface dipole tessellation, which are specifically important to model the electrical properties of the human head. As described below in Section 8, efficient construction of accurate electrical volume conduction head models is important to improve the source analysis of human electrophysiological activity with dense array technologies. Accurate head tissue conductivities and geometries are also important to optimize the transcranial current delivery to stimulate the brain that is now possible with dense array technologies.

In addition to these somewhat unique capabilities, BrainK implements certain tissue segmentation and surface extraction algorithms that are useful in medical image processing generally and that can be evaluated in comparison with existing software that has become well known in the neuroimaging research community. Examples of the software examined here are FreeSurfer, SPM, FSL, and BrainVisa. To assure accuracy for medical applications, validation of BrainK's unique algorithms against well-known reference software not only is scientifically useful but also forms an integral part of medical quality verification and validation. In addition, medical use requires that the functionality is fast and intuitive, such that BrainK must meet standards of efficiency and ease of use.

Our current evaluation work pays particular attention to brain tissue segmentation due to the fact that the accuracy of brain tissue segmentation is critical for a variety of medical and neurological applications such as source localization and cortical dysplasia detection.

### 4.1. Comparative Packages

Below is a brief description of the four brain image analysis tools used for comparative performance evaluation. Although these tools provide varying functionality in neuroimage analysis, they all support automatic T1-weighted human brain MR image segmentation. Our comparative evaluation is focused on the image segmentation task.

#### 4.1.1. FreeSurfer

FreeSurfer [29, 30] is a set of tools for reconstruction of cortical surfaces from structural MRI data and for the overlay of functional data onto the reconstructed surface. FreeSurfer is developed in the Nuclear Magnetic Resonance (NMR) Center, Massachusetts General Hospital. The cortical surface reconstruction pipeline in FreeSurfer mainly consists of three steps. First, a brain mask is extracted with alignment of the structure MR image to the Talairach Atlas and the bias field is corrected. Then, the brain volume is labeled as various cortical or subcortical structures in a procedure based on both a subject-independent probabilistic Atlas and subject-specific measured values. Finally, the cortical surfaces are constructed from the prior segmentation, which involves a topology correction procedure.

#### 4.1.2. SPM

SPM (Statistical Parametric Mapping) is a statistical technique for testing hypotheses about functional imaging data [31]. SPM also refers to the software developed by the Wellcome Department of Imaging Neuroscience, University College London, to carry out such analysis. SPM features structural MRI segmentation as well as a series of functional neuroimage analysis. Structural MRI segmentation in SPM can be characterized as a circular procedure that involves alternating three processing steps [32]: a bias correction step that corrects the intensity inhomogeneity, a registration step that normalizes the image to standard tissue probability maps, and a segmentation step that classifies image voxels into different tissue types. As the segmentation results, SPM assigns each image voxel three probabilities with respect to three tissue types: CSF, GM, and WM. Our experiments were conducted with SPM5, which is old with respect to the latest SPM12, but their implementation is based on the same algorithm presented in [32], although the newer version makes use of additional tissue classes and multichannel segmentation and incorporates a more flexible image registration component, as stated in the release notes of SPM12.

#### 4.1.3. FSL

FSL (the FMRIB Software Library) is a collection of functional and structural neuroimage analysis tools [33]. For structural segmentation, FSL applies the Brain Extraction Tool (BET) for segmenting brain from nonbrain regions in structural and functional data, and FAST (FMRIB's Automated Segmentation Tool) for bias field correction and brain segmentation into three tissue types: CSF, GM, and WM. Structural MRI segmentation in FSL consists of two steps: using BET to extract the brain and using FAST to classify tissue types. BET performs skull stripping with a surface model [34]. The underlying method of FAST is based on an expectation-maximization algorithm combined with a hidden Markov random field (MRF) model [35]. Due to the regularization of the MRF model, FAST is supposed to be more robust to noise than standard finite mixture model based methods.

#### 4.1.4. BrainVisa

BrainVisa [36, 37] is software developed at Service Hospitalier Frédéric Joliot (SHFJ) that encompasses an image processing factory and is distributed with a toolbox of building blocks dedicated to the segmentation of T1-weighted MR image. Structural MRI segmentation in BrainVisa consists of four main steps. First, the user prepares the data for segmentation by specifying several key landmark points including the anterior commissure (AC), the posterior commissure (PC), an interhemispheric point, and a left hemisphere point. A brain mask is then extracted including only white matter and gray matter integrating bias field correction [38] and histogram analysis [39]. This is followed by a hemisphere partition and removal of cerebellum with morphological image analysis [40]. Finally, cerebral gray matter and white matter are differentiated with histogram analysis [41].

## 5. Datasets

The evaluation was performed on three image datasets: a set of BrainWeb data with ground truth segmentation, a set of IBSR data with manually guided expert segmentation, and a set of real scans of subjects with either mild cognitive impairment or Alzheimer's disease.

### 5.1. BrainWeb

The BrainWeb dataset is a group of 8 realistic T1-weighted MR simulated images with ground truth segmentation provided by BrainWeb, a simulated brain database [42, 43]. All 8 MR images are simulated on a normal anatomical model. The resolution of the images is 1 mm^3^. In the ground truth image, all voxels in the image are segmented into the following tissue types: Background, CSF, GM, WM, Fat, Muscle/Skin, Skin, Skull, Glial Matter, and Connective.

A variety of noise levels and levels of intensity inhomogeneity (i.e., intensity nonuniformity (INU)) are artificially introduced in the simulated images, as listed in Table 1. As stated in BrainWeb documentation [44], the “noise” in the simulated images has Rayleigh statistics in the background and Rician statistics in the signal regions. The “percentage noise” number represents the percent ratio of the standard deviation of the white Gaussian noise versus the signal for a reference tissue. The noise reference tissue used in our dataset is white matter. The meaning of the intensity inhomogeneity level is as follows. “For a 20% level, the multiplicative INU field has a range of values of 0.90–1.10 over the brain area. For other INU levels, the field is linearly scaled accordingly (e.g., to a range of 0.80–1.20 for a 40% level).” According to BrainWeb, the INU fields are realistic in that they are slowly varying fields of a complex shape and were estimated from real MRI scans.

### 5.2. IBSR

The IBSR dataset is a group of 18 T1-weighted real MR brain datasets. Their manually guided expert segmentation is included in the Internet Brain Segmentation Repository (IBSR) supported by the Center for Morphometric Analysis (CMA) at Massachusetts General Hospital [45]. The slice resolution of all datasets is 1.5 mm and the XY resolution varies from 1 mm^2^ to 0.837 mm^2^. The MR images have been “positionally normalized” into the Talairach orientation, but all five tools performed on this group of data assumed that the images were not normalized. The MR images were also processed by the CMA bias field correction routines, but it is not guaranteed that the intensity inhomogeneity is completely corrected. All five tools therefore treated the datasets as if no bias field correction had been performed.

Each MR image was manually segmented into 44 individual structures including 3rd ventricle, 4th ventricle, Brainstem, and left and right: accumbens area, amygdala, amygdala anterior, caudate, cerebellum cortex, cerebellum exterior, cerebellum white matter, cerebral cortex, cerebral exterior, cerebral white matter, hippocampus, Inf Lat vent, lateral ventricle, pallidum, putamen, thalamus proper, ventral DC, and vessel.

The 18 MR images represent various levels of image quality. To organize the evaluation by general image quality, we divided the images into two subgroups: the first 13 MR images with good quality and 5 more MR images with bad quality. Note that the ordering of the IBSR datasets is different from the original order. A map of the order we used to the original order is “1, 2, 5, 6, 7, 8, 9, 10, 11, 12, 14, 15, 16, 17, 18, 3, 4, 13.” For example, when we refer to the 3rd dataset, it is actually the 5th in the original order.

### 5.3. Datasets Reflecting Neuropathology

In addition to the BrainWeb and the IBSR datasets, which were used for both quantitative and qualitative evaluation, we also tested five tools on an auxiliary group of 8 real MR images scanned from subjects with minor cognitive impairment or those with Alzheimer's disease. The resolution of these datasets is 1.139 × 1.211 × 1.211 mm^3^. The source of these datasets is the Neurobiology Research Unit [46] in the University Hospital Rigshospitalet in Denmark. No ground truth or manual segmentation is provided for these datasets; the issue for the evaluation is primarily whether the abnormal brains can be segmented with apparently reasonable accuracy.

## 6. Quantitative Evaluation

In this section, we present a quantitative evaluation on the segmentation accuracy, robustness, and computational efficiency of BrainK in comparison to other four packages. We use the widely used Dice metric [1, 4, 46, 47] as the measurement for segmentation accuracy. The standard deviation of the Dice metric provides a measure of segmentation robustness. Computational efficiency is measured as the required run time of each package.

### 6.1. Dice Metric

Let TP refer to the number of true positives, FP to false positives, and FN to false negatives. The Dice metric is then given by

(3) $$\begin{array}{c} Dice\;\;metric=\frac{2\times TP}{2\times TP+FP+FN}. \end{array}$$

TP, FP, and FN are measured versus the manual segmentation of real MR datasets or the ground truth of simulated images. Note that when the segmentation is given as a probability between 0 and 1 for each image voxel for each tissue class (such as in the case of SPM), then TP, FP, and FN are calculated as the sum of the probabilities instead of discrete counting.

For quantitative evaluation using Dice metric, we must decide the tissue type on which the metric is measured. BrainVisa only performs cerebrum segmentation while FSL and SPM segment the entire brain into CSF, GM, and WM without extraction of the cerebrum. FreeSurfer also performs segmentation on the whole brain, but it segments the brain into a greater number of tissue types (including cerebral white matter and cerebral cortex). Currently, BrainK performs segmentation on the entire image, and it is able to differentiate cerebrum from cerebellum and one cerebral hemisphere from the other. In our quantitative evaluation, we must calibrate the segmentation of the five packages within a standard framework, so that common tissue types can be used for quantitative metric measurements.

For the BrainWeb datasets, we calibrated the segmentation of five tools to the segmentation of cerebral WM and cerebral GM and measured the Dice metrics with respect to these two tissue types. To enable this, we manually partitioned the ground truth whole brain (WM plus GM) at the brainstem to extract the cerebral WM and the cerebral GM. Cerebral WM and cerebral GM also need to be extracted for the SPM and FSL segmentation results. We use a procedure (described in the next paragraph) that almost “perfectly” partitions the segmentation results based on the ground truth partition. For BrainVisa and BrainK, no transformation in the calibration is required. For FreeSurfer, we just need to relabel all cerebral cortex voxels and all subcortical voxels excluding cerebral WM as cerebral gray matter.

Let TP-Cerebrum and TP-Cerebellum, respectively, denote the set of true positives of cerebrum and cerebellum in the segmentation of SPM and FSL. Let FP-Brain denote the entire false positives including those in cerebrum and cerebellum. The partition of the brain segmented by FSL and SPM is essentially the partition of FP-Brain into false positives in cerebrum and those in cerebellum, which is described as follows. For each voxel *v* in FP-Brain, if it has a shorter path in FP-Brain to TP-Cerebrum than any paths in FP-Brain from *v* to TP-Cerebellum, then *v* is taken as a false positive (of WM or GM) in cerebrum; otherwise, it is taken as a false positive (of GM or WM) in cerebellum.

For the IBSR data, we calibrated the segmentation of five tools to the segmentation of cerebral cortex and cerebral WM and measured the Dice metrics with respect to these two tissue types. These quantitative metrics give an evaluation on the accuracy of the cortical surface reconstruction which depend on segmentation of cerebral cortex and cerebral WM and are irrelevant to segmentation of subcortical gray matter tissues. Since FreeSurfer explicitly labels cerebral cortex and cerebral white matter, we do not need to do any transformation in the calibration. The calibration of FSL and SPM first conducts the brain partition to extract the cerebral WM and cerebral GM. Given the set of cerebral WM and cerebral GM segmented by FSL, SPM, BrainVisa, or BrainK, we measured the Dice metrics with respect to cerebral cortex and cerebral WM in the way described below.

### 6.2. Experiment Settings

#### 6.2.1. FreeSurfer

We tested FreeSurfer on both the BrainWeb and the IBSR datasets in a fully automatic mode without any user intervention. An issue in collecting FreeSurfer segmentation results is the production of the cerebral cortex mask. There is a so-called “aseg” image and a “ribbon” image and both of these record voxels are labeled as cerebral cortex. The “ribbon” data is what FreeSurfer suggests to use, but it has more false negatives than the “aseg” data. In contrast, the “aseg” image is an intermediate result, and it has more false positives than the “ribbon” data. We applied a simple morphological closing operation on the union of the cortex ribbon and the subcortical structures so that certain true cerebral cortex voxels labeled in “aseg” but missed in “ribbon” are covered. This procedure apparently improved the performance of the cerebral cortex segmentation. The Dice metric for the “aseg” image was 0.7353, for the “ribbon” image was 0.8009, and for the “closed” image was 0.8212.

#### 6.2.2. FSL

In our first batch of experiments with FSL, we let FSL automatically extract the brain and performed brain tissue classification on both the BrainWeb and the IBSR datasets. However, FSL generated poor results on the brain extraction and brain tissue classification on 6 of the IBSR datasets (dataset 5 to dataset 10). In our second batch of experiments, therefore, we used different parameters in FSL, obtained better brain masks for these datasets, and then repeated the brain tissue classification. The brain masks generated in the second batch of experiments were still not good enough. We therefore used the brain masks generated by FreeSurfer for the brain tissue classification in FSL. This delivered the best FSL brain segmentation performance on the 6 difficult IBSR datasets. The three batches of experiments on FSL show that the brain extraction algorithm of FSL is not robust on the IBSR datasets, but the brain tissue segmentation itself performed well given good brain masks. The performance of FSL on the 6 IBSR datasets with respect to the three batches of experiments is shown in Table 2.

#### 6.2.3. SPM

In our first batch of experimental tests with SPM, we used the default parameters and let SPM automatically perform brain tissue segmentation on the BrainWeb and the IBSR datasets. In the second batch of experiments, we changed the parameter “bias regularization” from the default “very light regularization” to “medium regularization” and reran SPM on the IBSR datasets. SPM is supposed to be used with greater bias regularization when it is known* a priori* that there is less intensity inhomogeneity in the image. Since the IBSR datasets were processed with bias field correction, the use of “medium regularization” rather than the default “very light regularization” improved the performance of SPM on almost all IBSR datasets. For the cerebral cortex, the mean Dice metric over the 18 ISBR datasets was 0.7609 for very light regularization and 0.7656 for medium regularization. The mean Dice metric for white matter was 0.8572 for very light regularization and 0.8707 for medium regularization. We used the best regularization for each dataset in our comparative evaluation.

#### 6.2.4. BrainVisa

We tested BrainVisa on both the BrainWeb and the IBSR datasets automatically, with the exception that we manually specified landmark points including the AC point, the PC point, an interhemispheric point, and a left hemisphere point. BrainVisa produced an empty brain mask in the 9th IBSR dataset and was unable to generate brain masks for the 13th and the 18th datasets. In both cases, we set the Dice metrics to be 0.

#### 6.2.5. BrainK

BrainK was tested on the BrainWeb and the IBSR datasets fully automatically.

### 6.3. Comparison Results

#### 6.3.1. Segmentation Accuracy on the IBSR Datasets

We collected the Dice metrics with respect to cerebral cortex (Table 3) and cerebral WM (Table 4) using the five software packages on the IBSR datasets.

On average, BrainK performed best on cerebral cortex segmentation on all 18 IBSR datasets, good and bad, and on the 13 good datasets exclusively. In particular, BrainK's cerebral cortex performance is consistently better than the four other packages on the 13 good datasets except for the 4th dataset, where BrainK's performance is almost identical to the best, and the 12th dataset, where BrainK's performance is close to the best. The cerebral cortex segmentation performance of the five packages on the five bad datasets is similar, except that BrainVisa generated empty brain mask for the 18th dataset.

To provide an estimate of the statistical significance of the comparison of BrainK with the other packages, we conducted paired comparison *t*-tests across these 18 datasets. BrainK was more accurate at segmenting cerebral cortex with the ISBR datasets than BrainVisa (*p* < 0.02), SPM (*p* < 0.006), FreeSurfer (*p* < 0.0005), and FSL (*p* < 0.0009). If we omitted the three datasets on which BrainVisa failed, the comparisons again showed BrainK to be more accurate than BrainVisa (*p* < 0.002), SPM (*p* < 0.009), FreeSurfer (*p* < 0.001), and FSL (*p* < 0.002).

On average, FSL performed best on cerebral WM segmentation on the 18 IBSR datasets and on the 13 good datasets. However, BrainK's performance was very close to FSL's performance in this comparison. The performance of the five packages on the five bad datasets is similar, except that BrainVisa generated empty brain mask for the 18th dataset, and FreeSurfer gave particularly poor performance for the 14th dataset.

#### 6.3.2. Segmentation Robustness on the IBSR Datasets

We calculated the standard deviations of the Dice metrics on the IBSR datasets. Together with the mean Dice metrics, these values indicate the segmentation robustness of the five packages on MR images scanned from different subjects. Greater mean Dice metric and lower standard deviation indicate greater robustness with respect to segmentation accuracy.

Two groups of standard deviations were calculated: one on the total 18 IBSR datasets and one on the 13 good IBSR datasets. BrainK demonstrated the lowest standard deviation of the software packages with respect to cerebral cortex on both the total 18 IBSR datasets and the 13 good IBSR datasets, as shown in Table 5. The lowest mean and standard deviation Dice metric with respect to cerebral cortex indicate that BrainK demonstrates the best accuracy and robustness with respect to cerebral cortex on the IBSR datasets. For cerebral WM, BrainK and FSL performed very similarly with respect to both the mean and the standard deviation of the Dice metric on both the total 18 IBSR datasets and the 13 good IBSR datasets. BrainK and FSL tied for the best accuracy and robustness with respect to cerebral WM on the IBSR datasets. Considering both cerebral cortex and cerebral WM, these results suggest that, in general, BrainK demonstrated the best overall performance on the IBSR datasets.

#### 6.3.3. Segmentation Robustness with respect to Noise and IIH on the BrainWeb Datasets

As described in Section 3.1, the BrainWeb datasets vary in noise levels and intensity inhomogeneity (IIH) levels. The performance in Dice metrics of the five packages with respect to cerebral GM and cerebral WM is listed in Tables 6 and 7.

Among the five packages, FreeSurfer demonstrated the lowest performance variation over different noise levels. SPM and BrainVisa are similar in showing the highest performance variations over different noise levels. BrainK and FSL have medium performance variations over different noise levels, compared to the other three packages. Although FreeSurfer performed consistently over different noise levels, it also gave results with the lowest accuracy on average.

For each of the four noise levels, we also tested the packages on images with two different IIH levels. All five packages gave little variation over different IIH levels. The only exception is for BrainVisa with *N* = 9% and IIH = 40%. This is due to a poor brain mask.

It is worth noting that, in real MR scans, the intensity inhomogeneity may occur in various and unknown patterns. Such noise could occur together with other difficulties that may be not present in the simulated BrainWeb datasets. Therefore, we remark that our experiments with the BrainWeb dataset should not be taken to give a thorough and sufficient evaluation on the five packages with respect to the IIH robustness.

#### 6.3.4. Computational Efficiency

The execution times of the fives packages tested on the IBSR datasets and the BrainWeb datasets are listed in Table 8. The experiments were all run on a single 2.8 Ghz Intel Xeon processor. Among the five packages, BrainVisa took the least amount of time, but it also produced the lowest segmentation accuracy and robustness on the IBSR datasets. FreeSurfer is well known for long execution times. However, these long execution times cover segmentation of more subcortical structures as well as reconstruction of cortical surfaces. It should be noted that most of the BrainK time was spent for topology correction, which was not included in the execution times of the other three.

## 7. Qualitative Evaluation

In this section, we give a qualitative evaluation of the five packages based on the results of applying the packages to the IBSR datasets, the BrainWeb datasets, and the 8 pathological datasets with mild cognitive impairment or Alzheimer's disease. We first summarize and compare the segmentation functionalities of the five packages followed by the discussion of their automaticity. Finally, we present various segmentation abnormalities that we observed in the experiments.

### 7.1. Segmentation Functionalities

The following is a summarization and comparison on the main segmentation features of the five packages:

- * Bias Field Correction*. Freesurfer, SPM, FSL, and BrainVisa all integrate a bias field correction procedure, either prior to tissue classification or combined with the classification. BrainK, on the other hand, does not need an explicit bias field correction because the relative thresholding method is robust to bias field in arbitrary patterns.
- * Brain Extraction*. FSL, FreeSurfer, and BrainVisa provide separate tools for brain extraction (i.e., skull stripping) prior to brain tissue classification, whereas SPM combines brain extraction with tissue classification. BrainK, on the other hand, performs brain extraction after tissue classification. Note that the brain mask generated by BrainVisa is meant to contain only GM and WM while the brain mask generated by FSL and FreeSurfer is meant to contain CSF as well as GM and WM.
- * Tissue Classification*. FSL and SPM segment the brain volume into three tissue types: CSF, GM, and WM. BrainVisa extracts cerebral WM and cerebral GM. BrainK classifies the entire region of interest into CSF, GM, and WM, extracts the GM and WM, and separates cerebellum from cerebrum. BrainVisa and BrainK also provide cerebral hemisphere partition. FreeSurfer segments a whole brain into 37 individual structures including cerebral cortex, cerebral WM, a set of subcortical structures, brainstem, and cerebellar structures.
- * Cortical Surface Reconstruction*. BrainVisa, FreeSurfer, and BrainK support cortical surface reconstruction, but FSL and SPM do not. A core requirement for cortical surface reconstruction is to insure that the topology of the cortical surface is correct.
- * Multichannel Segmentation*. It is worth noting that FSL and SPM12 provide multichannel segmentation such that T1- and T2-weighted images can be combined together for increased accuracy. However, T2-weighted images are often not available and when they are available, T2-weighted images often decrease the contrast between grey and white matter and hence lead to bad performance in practice.

### 7.2. Segmentation Automaticity and User Intervention

All five packages support highly automatic brain segmentation, with little or no user intervention. FreeSurfer allows the user to start the cortical surface reconstruction without any intervention. In case the segmentation is not satisfactory, FreeSurfer supports interactive tools to allow the user to modify the brain mask and to add control points to improve the intensity normalization of WM, a procedure that is extremely important for the performance of FreeSurfer's whole brain segmentation. FreeSurfer also supports interactive tools for editing the final results generated by the automatic processing.

FSL also allows the user to start the segmentation without any intervention. In FSL, brain extraction and tissue classification are performed, respectively, by the BET and FAST algorithms. The BET performance substantially influences that of FAST. If the user is not satisfied with the brain extraction, FSL allows the user to select different parameters and rerun BET. However, our experiments with BET on the IBSR datasets and the pathological datasets show that BET cannot guarantee good brain extraction even with user intervention. FAST has custom options for the user to select whether to use the *k*-means segmentation or* a priori* probability maps for initial segmentation and to guide the *k*-means segmentation with manual intervention.

In SPM, brain segmentation can also be automatically started with the default parameters and SPM often generates good results. An important custom parameter of SPM is the one that controls the extent of bias field regularization. When any parameter is changed, the segmentation procedure has to be started over from scratch.

BrainVisa requires the user to prepare the data by first specifying several landmark points including the AC point, the PC point, an interhemispheric point, and a left hemisphere point. When the data is prepared by the user, BrainVisa automatically performs segmentation. BrainVisa supports interactive tools for the user to edit the segmentation results.

BrainK performs segmentation fully automatically, but the user also has the opportunity to tune the performance by changing key parameters such as the relative thresholds. Compared to the user intervention mechanisms in the other four packages, user intervention in BrainK is in the form of global parameter selection and has the following advantages. First, it is straightforward and requires little or no expertise to understand the meaning of the parameters and the criterion for selecting optimal ones. Second, it is very easy to operate by sliding a value bar. Third, it is very efficient and the user can obtain the effect of parameter selection in real time for such parameters as relative thresholds. Fourth, the parameters have global effect for segmentation and the user does not need to repeat similar operations for different local regions.

### 7.3. Segmentation Abnormalities

In examining the quality of the results, we first attempted to understand why BrainK consistently achieved better performance than the other packages with respect to cerebral cortex segmentation on the 13 good IBSR datasets [48]. We found that there was a “shrinking” effect on the cerebral cortex segmentation for FreeSurfer, FSL, SPM, and BrainVisa, which gives rise to a significant number of false negatives. This problem did not occur or was much milder in BrainK. We think the underlying reason is that BrainK uses a new image modeling mechanism that can adapt to wider variations of GM intensities, whereas the statistical methods used in other packages were misled by these intensity variations, resulting in missing many GM voxels that had lower intensities.

**Figure 9:**
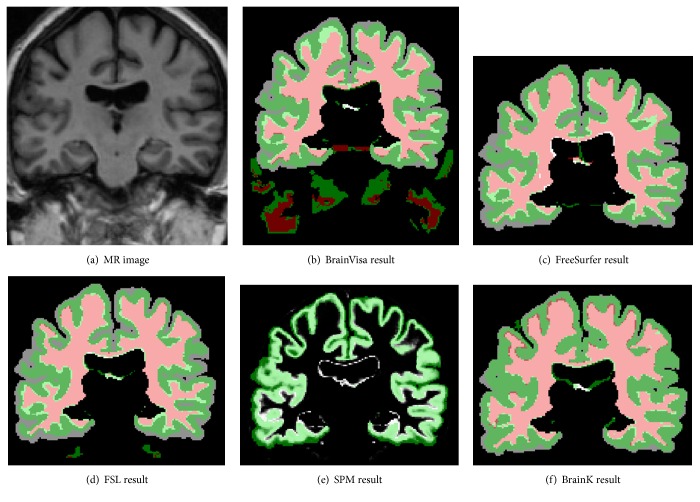
GM-shrinking phenomenon.

The contrasting cortex segmentation of the five packages is shown for a representative IBSR dataset in Figure 9. Pink stands for correct WM, red for false WM negative, light green for correct GM, very light green for false WM negative and false GM negative, dark green for false GM positive, and gray false for GM negative. Note that, for SPM, light green represents correct GM segmentation, darker green represents false GM negative, and gray level represents false GM positive.

**Figure 10:**
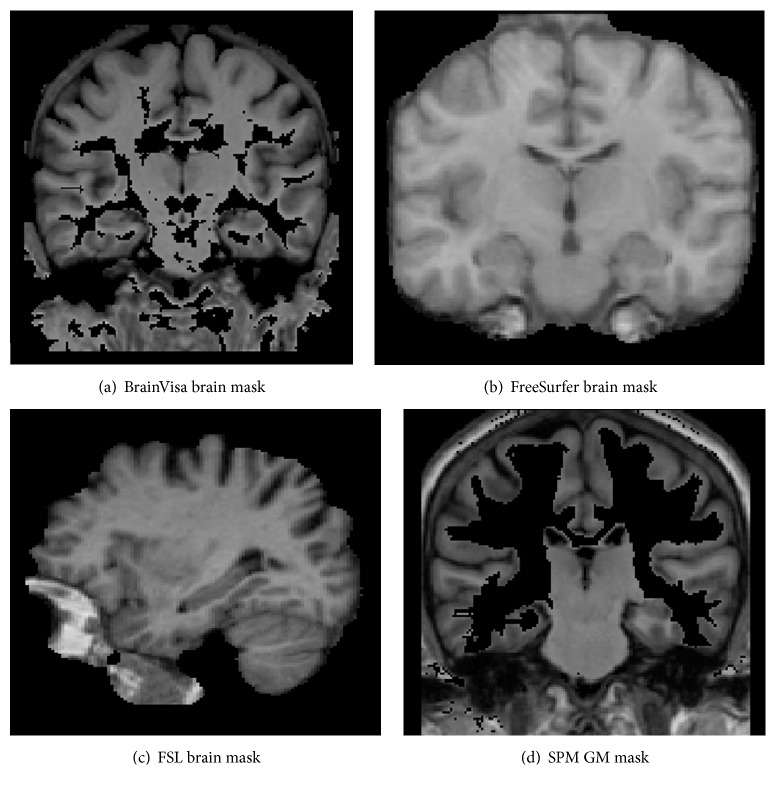
Poor brain masks.

**Figure 11:**
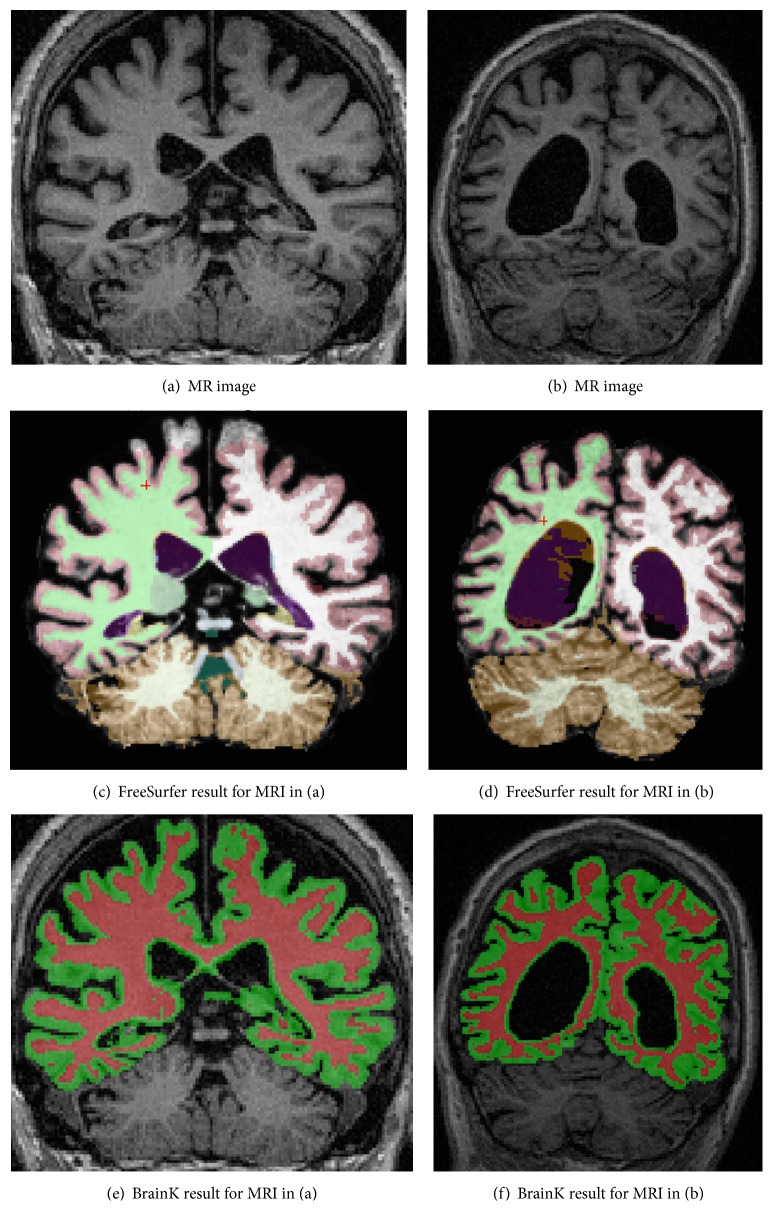
FreeSurfer abnormalities on the pathological datasets compared to BrainK.

A common problem in FSL, BrainVisa, and SPM is the poor brain extraction. Some examples are shown in Figure 10. Poor generation of the brain mask was the primary factor in the very poor performance for SPM and BrainVisa on the IBSR datasets. Since we used the considerably better brain masks generated by FreeSurfer for the FSL analysis, the FSL analysis is not representative. FreeSurfer did not encounter poor skull stripping, but an inaccurate brain mask may still be generated, as shown in Figure 11(b), where some nonbrain voxels with high intensities are taken as brain tissues. BrainK, on the other hand, does not depend on a brain extraction preprocessing step and robustly generated clean cerebrum masks as the union of the cerebral white matter and the cerebral gray matter on all tested datasets.

**Figure 12:**
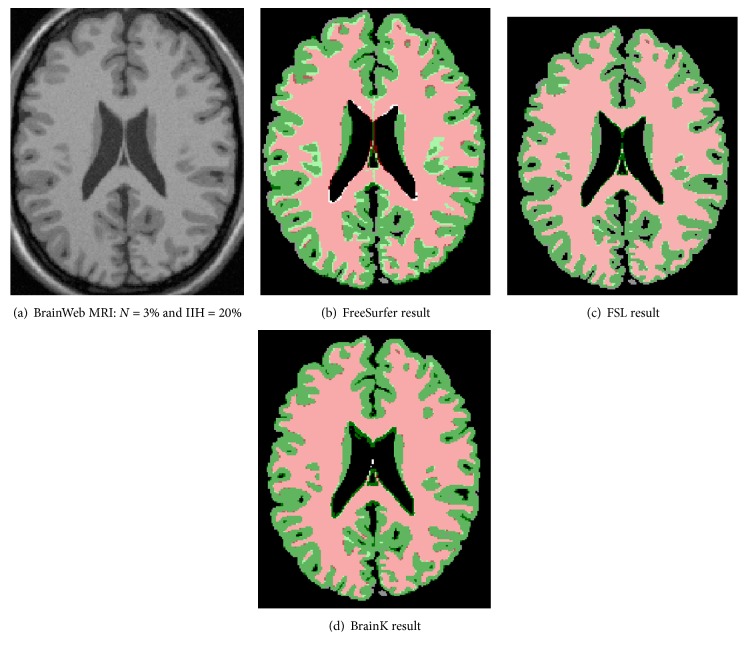
BrainK segmentation on the BrainWeb MRI compared to FreeSurfer and FSL.

We also found some other interesting abnormalities with FreeSurfer, as shown in Figure 11. For example, in Figure 11(c), FreeSurfer cut off a significant amount of cerebral WM and cortex at the top of the brain. In Figure 11(d), FreeSurfer was unable to segment the complete lateral ventricle of the subject with Alzheimer's disease. In Figure 12, FreeSurfer generated poor GM/WM segmentation even for a simulated image with excellent quality (noise level is 3% and IIH level is 20%) while BrainK and FSL generated excellent results. These abnormalities, we believe, are due to the overregularization of the* a priori* probability maps used in FreeSurfer.

## 8. Applications

At this point, BrainK has built functional electrical head models for more than 200 persons. Critical evaluation of BrainK's performance for generating segmented volumes and cortical surfaces continues in the applications of the* Modal Image Pipeline*, EGI's commercial software that incorporates the BrainK algorithms and workflows. Validation tests of physical tissue boundaries are conducted in the routine workflow for each head model construction. A first step in the FDM electrical lead field generation [49, 50], EGI's head physics code, is to test whether there are physically realistic segmentation properties in the BrainK output required for accurate electrical modeling. For example, there must be at least one voxel of CSF between the outer gray matter of the cortex and the inner table of the skull.

**Figure 13:**
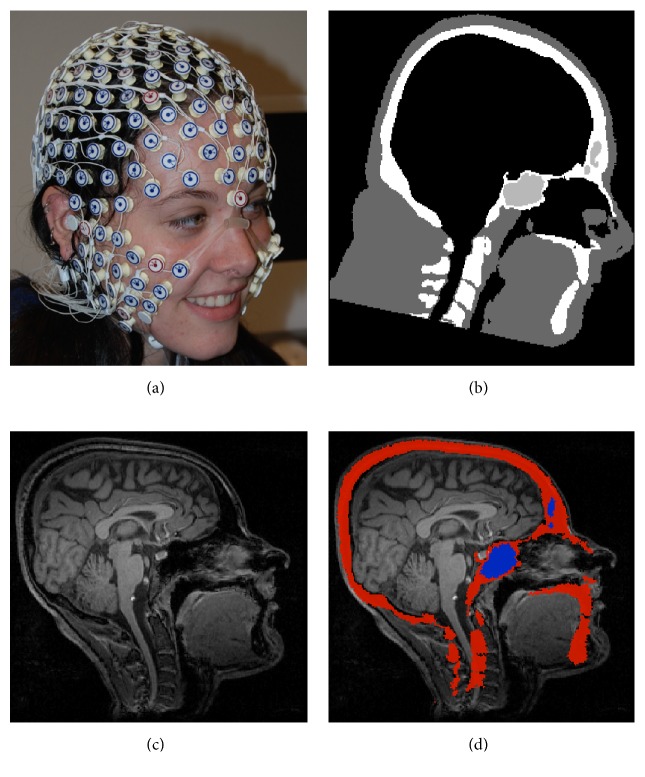
Skull Atlas-to-MRI registration with extensive warping of the skull to fit. (a) A volunteer in a 256 Net. (c) This person's MRI. (b) A database Atlas skull with a different shape. (d) Warp of the Atlas database skull to the volunteer's MRI.

There have been many applications of BrainK in research and development projects that have tested its capabilities and limitations. For example, although the skull Atlases are organized by age and gender in a database to minimize the warping required to fit an individual's MRI, tests have been conducted with skull CTs and MRIs of divergent shape (Figure 13), with good results.

**Figure 14:**
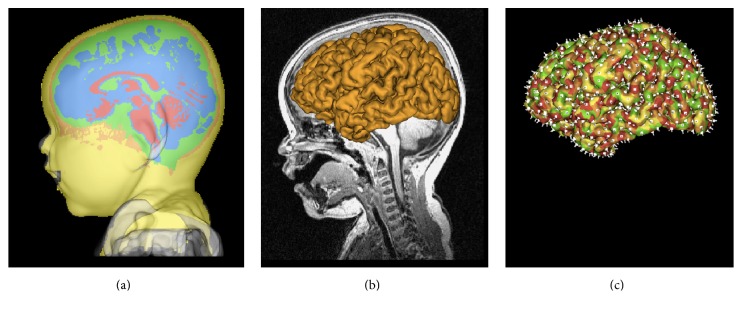
Electrical head model for an infant. (a) Registration of skull from CT with segmented MRI. (b) Cortical surface extraction overlaid with MRI. (c) Dipole tessellation of cortical surface for electrical source analysis.

Electrical source analysis with children requires age-appropriate electrical head models. Infants particularly require unique models because of their small head size and highly conductive skulls. BrainK has proven effective in adapting to the unique challenges of processing infant MRIs and CTs.  Figure 14 shows results from a current project to create pediatric electrical head models directed by Sergei Turovets at EGI and by Linda Larsen-Prior and Fred Prior at University of Washington St. Louis.  Figure 14(a) shows a normal infant's segmented MRI, with that infant's CT, registered to the segmented MRI, showing the thin skull and fontanel anterior to the vertex.  Figure 14(b) shows the extracted cortical surface, and Figure 14(c) shows the dipole tessellated cortical surface, prepared for electrical source analysis.

**Figure 15:**
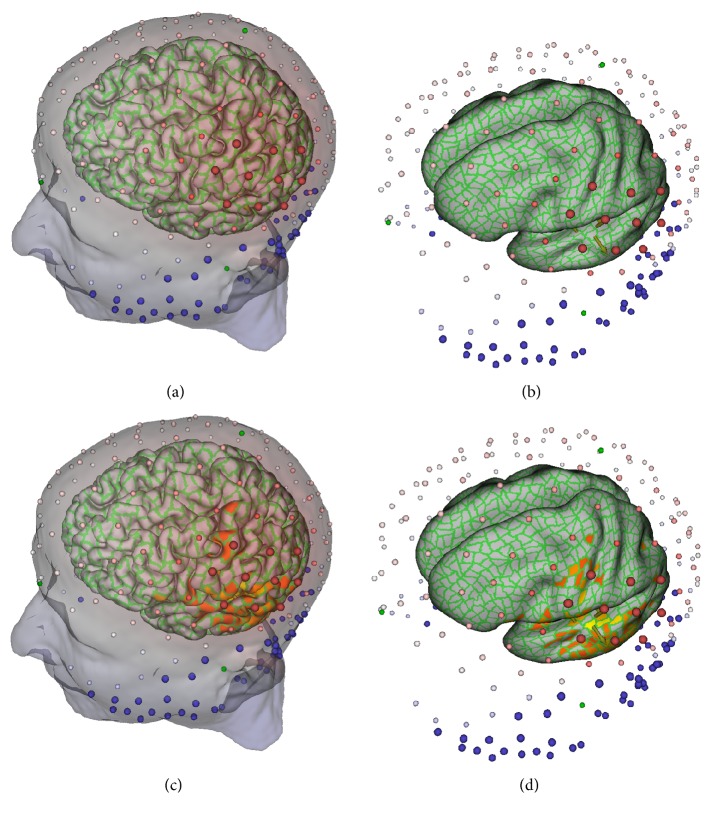
((a), (b)) Using BrainK's anatomy in the reciprocity visualization environment software to show the electrical field at the head surface (positive potential at red electrodes and negative potential at blue electrodes) created by electrical activity in auditory cortex (dipoles shown as gold arrows seen best in the inflated cortex at (b)). ((c), (d)) Through reciprocity theory, the field pattern of the cortical sources can be used to estimate the optimal electrodes (sources: red, sinks: blue) to impress electrical current on the auditory cortex for neuromodulation. Here, the impressed cortical current density is imaged with a hot metal palette (gold, orange, and yellow), thresholded at 50% of maximum.

The FDM electrical head models created by BrainK allow accurate estimation of the delivery of current, as well as its measurement, from the dense electrode array. With BrainK's anatomical results providing the geometry for the head physics code (Poisson equations for electromagnetic propagation in a finite difference model or FDM), lead fields are generated in the Modal Image Pipeline head physics code, describing the propagation from cortical sources to head surface electrodes (for EEG analysis) and from head surface electrodes to cortical sources (for transcranial electrical neuromodulation). The results are visualized on BrainK's anatomy and registered electrode positions in the reciprocity engine developed by Erik Anderson of EGI (Figure 15). In this illustration, cortical sources were chosen in the approximate region of auditory cortex, with the equivalent dipole for each cortical patch visualized with a gold arrow (seen best in Figure 15(b)). The lead field computation with the head physics model then shows the resulting head surface fields in the color of both the scalp surface and the electrodes (red is positive; blue is negative). Given this generated surface field, the reciprocity theorem states that impressing current on those cortical sources can be accomplished by applying current in the same surface pattern (red for anodal and blue for cathodal). The lead field from the head physics code is again used to estimate the current density that is impressed on the cortex by electrical neuromodulation with this electrode pattern (Figures 15(c) and 15(d)). The accuracy of the electrical solutions is fully dependent on the detailed geometry provided by BrainK, including not only the accurate (<1 mm) registration of sensors to the head surface, but also the specification of the orientation of the cortical patches provided by BrainK's characterization of the cortical surface.

## 9. Conclusion

BrainK is a software package that implements certain tissue segmentation and surface extraction algorithms that are useful in scientific and medical image processing. It also applies certain algorithms, such as skull image registration and cortical surface dipole tessellation, which are specifically important to model the electrical properties of the human head. Accurate electrical head models are proving important for improving the source analysis of human electrophysiological activity with dense array technologies and for optimizing transcranial current delivery to modulate brain activity.

## Figures and Tables

**Table 1:** Noise levels and IIH levels of the BrainWeb datasets.

Dataset	1	2	3	4	5	6	7	8
Noise level	3%	3%	5%	5%	7%	7%	9%	9%
IIH level	20%	40%	20%	40%	20%	40%	20%	40%

**Table 2:** Dice metrics collected for FSL using different brain masks.

Brain masks	Tissue types	Dice metrics on 6 IBSR datasets					
		5	6	7	8	9	10
Default FSL Brain mask	Cerebral cortex	0.6591	0.6806	0.7268	0.6887	0.7713	0.6762
	Cerebral WM	0.8845	0.8928	0.8891	0.8335	0.9095	0.8792

Customized FSL Brain mask	Cerebral cortex	0.7608	0.7735	0.7772	0.7740	0.7854	0.7859
	Cerebral WM	0.8722	0.8747	0.8766	0.8711	0.8953	0.8767

FreeSurfer Brain mask	Cerebral cortex	0.7312	0.7559	0.7898	0.7587	0.8277	0.7471
	Cerebral WM	0.8862	0.8953	0.8912	0.8914	0.9146	0.9009

**Table 3:** Dice metrics of five tools with respect to cerebral cortex on the IBSR datasets.

IBSR datasets	Dice metrics with respect to cerebral cortex				
	BrainVisa	SPM5	FreeSurfer	FSL	BrainK
1	0.7461	0.7705	0.8039	0.7803	**0.8682**
2	0.7953	0.8048	0.8175	0.8121	**0.8619**
3	0.7674	0.8080	0.8362	0.8361	**0.8714**
4	0.7233	0.8356	**0.8641**	0.8028	0.8612
5	0.2875	0.4621	0.7794	0.7312	**0.8638**
6	0.6610	0.6609	0.8068	0.7559	**0.8441**
7	0.7108	0.7065	0.7888	0.7898	**0.8638**
8	0.6982	0.7670	0.7800	0.7587	**0.8790**
9	0	0.7595	0.8128	0.8277	**0.8700**
10	0.7707	0.6868	0.7729	0.7471	**0.8611**
11	**0.8688**	0.8396	0.8702	0.8833	0.8634
12	0.8596	0.8541	0.8458	0.8582	**0.8772**
13	0	0.8588	0.8647	0.8554	**0.8673**
14	0.8406	0.8426	0.8065	**0.8429**	0.8315
15	0.8441	0.8439	**0.8487**	0.8381	0.8457
16	0.8260	0.8263	0.8413	**0.8426**	0.8281
17	**0.8445**	0.8443	0.8076	0.8278	0.8379
18	0	**0.8539**	0.8337	0.8278	0.8379
Mean	0.6247	0.7656	0.8212	0.8121	**0.8557**
Mean on 13 good datasets	0.6068	0.7365	0.8187	0.8030	**0.8656**

**Table 4:** Dice metrics of five tools with respect to cerebral WM on the IBSR datasets.

IBSR datasets	Dice metrics with respect to cerebral WM				
	BrainVisa	SPM5	FreeSurfer	FSL	BrainK
1	0.8652	0.8927	0.7964	**0.8971**	0.8789
2	0.8899	0.8940	0.8208	**0.9160**	0.8926
3	0.8649	0.8936	0.8138	**0.9084**	0.8874
4	0.8596	0.9013	0.8489	**0.9168**	0.9070
5	0.4097	0.7312	**0.9240**	0.8862	0.9101
6	0.7970	0.7422	**0.9115**	0.8953	0.8954
7	0.8255	0.8734	**0.9147**	0.8912	0.9001
8	0.8110	0.8923	**0.9203**	0.8914	0.9170
9	0	0.9059	0.9179	0.9146	**0.9224**
10	0.8457	0.8744	**0.9069**	0.9009	0.8790
11	0.8975	0.8927	0.8711	**0.9142**	0.8969
12	0.8858	**0.9014**	0.8099	0.8988	0.9006
13	0	**0.8955**	0.8647	0.8673	0.8748
14	0.8613	**0.8678**	0.7824	0.8632	0.8622
15	0.8541	0.8880	**0.8746**	0.8743	0.8637
16	0.8792	**0.8948**	0.8479	0.8933	0.8713
17	**0.8619**	0.8551	0.8592	0.8585	0.8463
18	0	**0.8764**	0.8124	0.8323	0.8463
Mean	0.6893	0.8707	0.8610	**0.8900**	0.8862
Mean on 13 good datasets	0.6886	0.8685	0.8708	**0.8999**	0.8971

**Table 5:** Standard deviation of Dice metrics of five tools on the IBSR datasets.

Sample groups		Standard deviations				
Datasets	Tissue type	BrainVisa	SPM5	FreeSurfer	FSL	BrainK
All IBSR datasets	Cerebral cortex	0.3155	0.1284	0.0308	0.0429	0.0192
	Cerebral WM	0.3351	0.0505	0.0473	0.0230	0.0225

13 good datasets	Cerebral cortex	0.3047	0.1414	0.0345	0.0476	0.0087
	Cerebral WM	0.3305	0.0593	0.0482	0.0143	0.0147

**Table 6:** Dice metrics of five tools with respect to cerebral GM on the BrainWeb datasets.

BrainWeb datasets		Dice metrics with respect to cerebral GM				
Noise level	IIH level	BrainVisa	SPM5	FreeSurfer	FSL	BrainK
3%	20%	**0.9292**	0.9173	0.8333	0.9242	0.9084
	40%	0.9247	0.9189	0.8342	**0.9268**	0.9086

5%	20%	**0.9197**	0.8989	0.8323	0.9193	0.8908
	40%	**0.9201**	0.8998	0.8323	0.9193	0.8858

7%	20%	0.8628	0.8673	0.8320	**0.9113**	0.8816
	40%	0.8740	0.8713	0.8312	**0.9127**	0.8827

9%	20%	0.8166	0.8255	0.8259	**0.8996**	0.8658
	40%	0.7836	0.8301	0.8264	**0.9019**	0.8678

**Table 7:** Dice metrics of five tools with respect to cerebral WM on the BrainWeb datasets.

BrainWeb datasets		Dice metrics with respect to cerebral WM				
Noise level	IIH level	BrainVisa	SPM5	FreeSurfer	FSL	BrainK
3%	20%	0.9550	0.9471	0.8849	**0.9672**	0.9558
	40%	0.9599	0.9533	0.8889	**0.9664**	0.9593

5%	20%	0.9552	0.9314	0.8824	**0.9567**	0.9494
	40%	0.9534	0.9315	0.8863	**0.9581**	0.9476

7%	20%	0.9325	0.8978	0.8779	**0.9448**	0.9382
	40%	0.9311	0.9008	0.8796	**0.9467**	0.9370

9%	20%	0.8926	0.8656	0.8757	**0.9332**	0.9296
	40%	0.8748	0.8701	0.8740	**0.9354**	0.9289

**Table 8:** Computation times of five tools on the IBSR and the BrainWeb datasets.

Datasets	Computation times					
	BrainVisa	SPM5	FreeSurfer	FSL	BrainK	BrainK (topology correction)
IBSR	1.5 m	34 m	27.2 h	5 m	17 m	14 m
BrainWeb	1.6 m	20 m	24.5 h	9 m	21 m	18 m
